# IgM memory B cells: a mouse/human paradox

**DOI:** 10.1007/s00018-012-0971-z

**Published:** 2012-04-06

**Authors:** Claude-Agnès Reynaud, Marc Descatoire, Ismail Dogan, François Huetz, Sandra Weller, Jean-Claude Weill

**Affiliations:** 1 Faculté de Médecine, Site Necker-Enfants Malades, INSERM U783 “Développement du système immunitaire”, Université Paris Descartes, 156 rue de Vaugirard, 75730 Paris Cedex 15, France; 2 Unité de Biologie des Populations Lymphocytaires, CNRS URA 1961, Institut Pasteur, 25 rue du Docteur Roux, 75724 Paris Cedex 15, France

**Keywords:** B cell memory, Somatic hypermutation, AICDA, Marginal zone B cells

## Abstract

Humoral memory is maintained by two types of persistent cells, memory B cells and plasma cells, which have different phenotypes and functions. Long-lived plasma cells can survive for a lifespan within a complex niche in the bone marrow and provide continuous protective serum antibody levels. Memory B cells reside in secondary lymphoid organs, where they can be rapidly mobilized upon a new antigenic encounter. Surface IgG has long been taken as a surrogate marker for memory in the mouse. Recently, however, we have brought evidence for a long-lived IgM memory B cell population in the mouse, while we have also argued that, in humans, these same cells are not classical memory B cells but marginal zone (MZ) B cells which, as opposed to their mouse MZ counterpart, recirculate and carry a mutated B cell receptor. In this review, we will discuss these apparently paradoxical results.

## Introduction

The T-dependent B cell response starts, after encounter of antigen and appropriate cognate T cell help, by a germinal center reaction involving a strong proliferative phase. Expression of activation-induced cytidine deaminase (AID) during this reaction allows a series of modifications of the BCR, including mutations of the variable regions of the immunoglobulin genes and isotype switch, shifting the surface Ig expression from IgM to IgG and IgA. The GC reaction peaks in the second week, with both IgM^+^ and IgG^+^ centroblasts detectable, and wanes after a few weeks [1]. Selection of B cells displaying enhanced antigen recognition takes place in the GC environment, mediated by antigen–antibody complexes retained by the follicular dendritic cell network that structures germinal centers. This selection results in the generation of long-lived plasma cells and memory B cells with an affinity matured, mutated BCR.

When working on B cell memory, obvious questions come to mind which, ironically enough, have been around more or less in the same terms for the last 30 years.

### What is the role of antigen in maintaining memory?

It has been proposed by several authors that memory B cells are maintained by the presence of their cognate antigen [2–4] and that they could accordingly be continuously stimulated giving rise to short-lived plasma cells that would contribute to maintain the level of specific antibodies in the serum [5]. Conversely, it was shown by the group of K. Rajewsky, through an experimental setting allowing memory B cells to acquire a new antigenic specificity that memory B cells could survive in a quiescent state in the absence of antigen [6, 7]. Although this question has never been approached systematically, it has been inferred that certain antigens such as viral particles may persist in the organism and entertain long lasting GCs [4].

### How do memory B cells respond to a re-encounter with their cognate antigen?

In most reports, the boost is performed a few weeks after the priming and triggers an active GC reaction and the formation of antibody-forming cells (AFC) [8]. However, in some other settings, AFCs could be observed after a boost without the formation of GCs [9–11]. Here again, the analysis of the different protocols used may allow us to explain these conflicting data.

### How long does B cell memory last?

While this topic has been thoroughly studied in humans, few data are available concerning this question in mice, as sensitive enough models have been developed only recently [10, 12]. Assuming that the persistence of certain antigenic structures used for immunization must not be different in mice and men, it remains striking to see such a difference in the duration of the B cell memory response between the two species (around 12 months for mice and decades for humans).

We will at first describe the model we have established to follow B cell memory in the mouse and confront the results obtained with recent data from other groups, with the aim to integrate these different models into a plausible framework. In the second part, recent data on human IgM^+^IgD^+^CD27^+^ B cells will be discussed, confronting the germinal center versus marginal zone models for their ontogeny and function.

## IgM memory B cells in the mouse: phenotype and function

### Two layers of IgM and IgG memory in mice with different effector functions

In order to monitor the fate of B cells in a physiological situation, we have established a mouse model allowing to timely and irreversibly mark B cells as they engage in a germinal center reaction during a T-dependent response [12]. This was achieved by inserting the tamoxifen inducible Cre-ERT2 gene at the AID locus, allowing its expression in centroblasts during the GC reaction. After backcrossing with the Rosa26-LoxP-EYFP reporter mouse, injection of tamoxifen coupled with immunization induced the expression of EYFP in the responding cells. Two antigens have been used, one in a particulate form, sheep red blood cells (SRBC), and one in a soluble form, NP-CGG precipitated with alum, with both antigens injected intra-peritoneally.

This highly sensitive model, which allowed the follow-up of memory B cells generated in a precise immune response for up to 1 year by flow cytometry and confocal microscopy, revealed new features of B cell memory.

Active germinal centers identified by GL7 or PNA staining and containing proliferating IgM^+^ and IgG1^+^ centroblasts could be observed for up to 8–10 months after two immunizations with SRBC. These centroblasts were embedded in FDCs in contact with CD4 T cells [12]. Conversely, germinal centers waned after 2–3 months after two immunizations with NP-CGG, as shown previously [13].

**Fig. 1:**
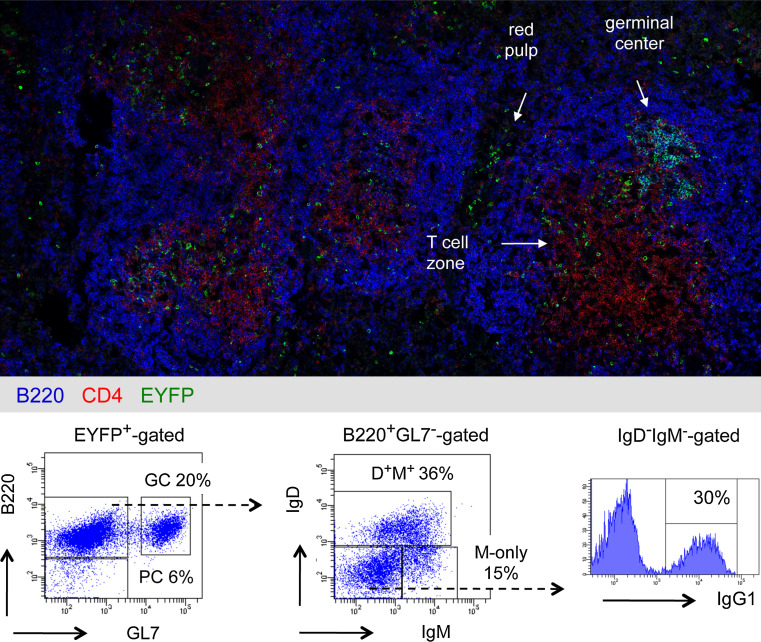
Location and phenotype of memory B cell subsets in the mouse. Transgenic mice bearing the Cre-ERT2 gene inserted at the AID locus and the ROSA26-loxP-EYFP reporter gene were immunized twice with SRBC at a 1-month interval, with simultaneous ingestion of tamoxifen [12]. (*Upper part*) Analysis by confocal microscopy of a mouse spleen, 2 months after the second SRBC immunization, using anti-B220, CD4 and EYFP antibodies, localization of EYFP^+^ cells in germinal centers, T cell zone and red pulp is highlighted. Very few EYFP^+^ cells are detectable in the marginal zone that forms the outer ring around B cell follicles. (*Lower part*) Flow cytometry analysis of EYFP^+^-cells for the markers B220, GL7 (germinal centers, GC), IgM, IgD and IgG1, plasmocytes (PC) being identified as B220^−^. Reported to the initial EYFP^+^-gate, the IgG_1_^+^ population, which includes most of the SRBC-specific switched cells, represents 12 % of the initial population

Memory B cells were observed up to 12 months after immunization with SRBC and were composed of two subsets, an IgM^+^ and an IgG^+^ subset. The IgM subset comprised IgM^+^IgD^+^ and IgM-only sub-populations. The IgG subset comprises IgG_1_ cells, which represent the major fraction of SRBC-specific switched memory B cells, as well as other isotypes (Fig. 1). Memory B cells and plasma cells were located within the spleen in the T cell zone and in the red pulp, with some cells in the follicles and very few in the marginal zone (Fig. 1).

Boosting the animal 12 months after two immunizations with SRBC induced the expansion of all the subsets with the more pronounced formation of GC B cells and plasma cells. The animals immunized with NP-CGG displayed an IgM^+^ and an IgG^+^ memory subset 3–4 months after the two immunizations while their response to a boost, 1 year after, only induced a small plasma cell response.

Transfer of the different memory subsets 2–6 months after SRBC immunization in normal, pre-immunized animals gave strikingly different results. The IgM^+^ subset, whether GL7^+^ or GL7^−^, gave rise to centroblasts, with part of them switching to IgG_1_, and to a small proportion of SRBC-specific IgM^+^ plasma cells. The IgG_1_ subset, whether GL7^+^ or GL7^−^, gave rise to SRBC-specific IgG_1_ plasmocytes with some maintenance of the IgG_1_ memory pool.

There were on average three and six mutations per sequence in IgM and IgG memory B cells, respectively (a mutation frequency determined on a 440 bp J_H_4 intronic sequence) [12]. This frequency of mutations did not change much between 2 and 6 months, implying, as previously shown, that the memory pool is made for a large part during the peak of the GC reaction [14]. On the contrary, IgM^+^ and IgG^+^ centroblasts showed an increasing amount of mutations with age, with some IgG sequences displaying up to 50 mutations at 6 months. Clonal relationships were found between the different subsets, with some large expansions of clones in the centroblast population.

In summary, this model revealed the existence of a long-lived IgM^+^ memory subset in addition to the classical IgG^+^ one. It also uncovered a division of labor involving these two subsets somehow similar to the one observed for memory T cells [15]. Upon a boost, IgM memory B cells maintained the central memory pool by going back to GC, undergoing new rounds of SHM and producing later on a new batch of IgM and IgG memory B cells. IgG memory B cells, on the other hand, behaved as effector memory cells giving rise to antibody secreting cells, with some self-maintenance capacity. In such sense, both central and effector memory B cell pool were replenished after a boost in preparation of a new encounter with the original antigen. The nature of the antigen also revealed clear differences. With a particulate antigen like SRBC, a protracted GC response, albeit with a reduced activity, was observed, and the mice gave a full response mobilizing all subsets 1 year after immunization. With a soluble protein like NP-CGG, GCs disappeared after 2–3 months and there was only a small plasma cell response after 1 year with no new formation of GCs (see below).

Overall, this model seems to suggest that particulate antigens give rise to a more durable B cell memory. Moreover, one could speculate that the centroblasts in persistent GCs, in which ongoing mutation is taking place, could feed the memory pool at a low level, giving rise to a much larger panel of antibody affinities and specificities against the original antigen. If one thinks in terms of a mutating pathogen, such diversity might help to maintain protection of the host. Assuming that GCs are structures open to circulating B cells [16], persistent GCs could also constantly recruit newcomers into the immune response. On the negative side, the large expansion of some clones observed in persisting GCs might represent an oncogenic threat.

### IgM memory B cells: controversial issues on effector functions

Confronting recent publications on B cell memory reveals striking similarities and differences. M. Shlomchik and his colleagues, using a mouse model in which NP^+^ memory B cells from an anti-NP V_H_ knocked-in mouse were transferred into an Ig transgenic animal with an irrelevant Ag specificity and boosted, confirmed the presence of an IgM and IgG memory subset. Moreover, they identified among these subsets new markers (PD-L2, CD80, CD73) whose combinatorial expression indicated a gradient of memory-like properties [17]. Along the same line, M. Jenkins and colleagues, using for the primary immunization the soluble protein phyco–erythrin (PE) precipitated with alum, also observed long-lived IgG and IgM memory subsets identified through the binding of this fluorophore [10]. Upon a boost, by injection of a large PE dose in emulsion with complete Freund’s adjuvant 1 year after the primary immunization, PE-specific IgG^+^ memory B cells expanded considerably and differentiated into PE-specific antibody producing cells. Strikingly, while the IgM^+^ memory subset was more prominent and more stable than its IgG^+^ counterpart, being present at a steady level until 450 days after immunization, it did not react to this robust antigen boost, and could only give rise to active GCs containing IgM^+^ and IgG^+^ centroblasts when transferred into a naive animal. As expected, the IgG memory subset gave rise 5 days after its transfer to switched memory B cells and plasmocytes whose secreted antibodies prevented the formation of endogenous GCs in the host. These authors concluded that the long-lasting IgM memory subset was inhibited by circulating anti-PE antibodies and hence represented a low affinity memory B cell pool that could serve as a reservoir of memory B cells once the level of circulating antibodies has declined [10]. The phenomenon of serum inhibition of a B cell response has been reported some time ago, the most likely explanation being that specific high affinity antibodies prevented the immunogen from gaining access to the B cell receptor [18]. A similar expansion of PE-specific antibody producing cells in the absence of GCs induction, was observed by R. Noelle and colleagues [11], who used the same PE antigen for the primary immunization and a minimal amount of soluble antigen for the boost, several weeks later. Surprisingly, a similar epitope masking phenomenon had been also reported during a T-independent response in which it was shown that memory B cells were in fact present but prevented from responding by the antigen-specific IgG antibodies [19].

In summary, for a soluble protein antigen, a boost performed either with a very low amount of antigen at a short interval after the primary immunization or more potently at a later time will not mobilize IgM memory B cells and therefore not induce a GC reaction. It will do so if the boost is more robust and not too distant from the primary immunization (6–8 weeks). In all cases, the boost will induce the differentiation of IgG memory B cells into AFCs. With particulate antigens such as virus particles or SRBC, IgM memory B cells will respond to a boost distant from the primary immunization even once GCs have mostly disappeared.

Different parameters can thus control the behavior of the IgM memory subset upon a booster immunization: the time elapsed between the primary immunization and the boost, and the dose and type of antigen used. It has been reported that a different signaling is required to trigger IgM and IgG memory B cells, both membrane-bound IgM and IgD bearing a short cytoplasmic tail of 3 amino-acids compared to the 28 amino-acid tail of membrane-bound IgG [20]. Effectively it was shown that phosphorylation of a tyrosine in the IgG tail recruited the Grb2 protein, which was responsible for the enhanced signaling observed upon antigen stimulation [21]. Moreover, another segment of the IgG tail seemed to favor oligomerization of the BCR thus increasing the recruitment of the Syk kinase and the calcium response [22]. It appears therefore that, for soluble proteins that often behave as monovalent antigens, circulating antibodies will more easily compete out the BCR at the surface of IgM memory B cells. The differential response observed with time for the IgM memory subset could be related to the presence of a residual GC reaction that would allow IgM memory B cells to be mobilized faster and to benefit from more potent local help from T helper follicular cells.

In conclusion, and as opposed to what has been proposed for T cells [23], the initial signaling event sustained by the B cell may not play such a decisive role in the generation of memory, taking into consideration that both soluble protein and particulate antigens will induce the formation of long-lived IgM and IgG memory subsets. The protocol of immunization and the type and dose of antigen and adjuvant used would seem more important for the efficiency of the memory response.

### Long-term memory in humans

**Fig. 2:**
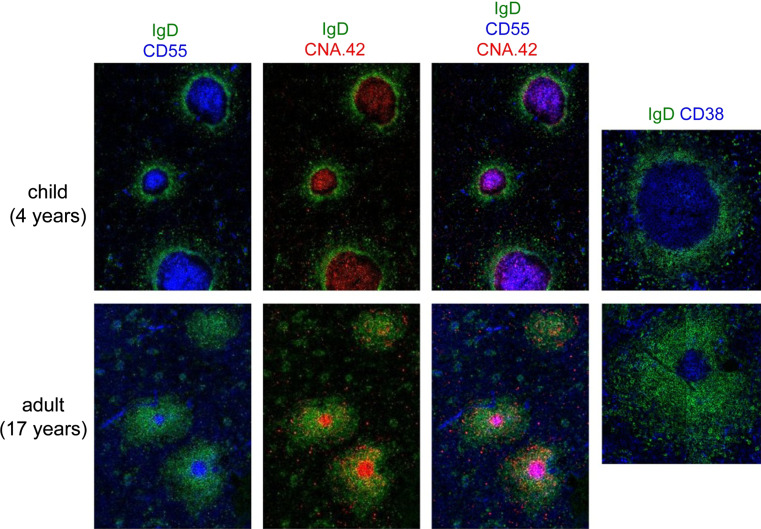
Active and residual germinal centers in human spleen. Germinal centers were analyzed by confocal microscopy of two spleen samples, one from a 4-year-old child, one from a young adult. The IgD marker was used, together with CNA.42 that marks follicular dendritic cells (FDC) throughout their development, and CD55 that marks FDCs in reactive lymphoid tissues [59]. CD38 marks GC B cells. Residual GCs can be found at a low frequency in the spleen of young adults, whereas germinal centers are always numerous and prominent in spleen samples from young children

How is this relevant to the human model, which, despite its obvious difficulties, offers a unique window of observation for long-term responses? Adult spleen rarely displays germinal centers, confirming that this organ at the adult stage is seldom engaged into active T-dependent immune responses. Conversely, a few residual GCs with a clear CD38^+^ B cell population within B cell follicles could be observed in the spleen of young adults, which evoked the mark of previous immune responses, due to vaccination or infections (Fig. 2) [24]. B cell memory against certain vaccines such as smallpox and yellow fever can have an almost infinite lifespan [25–27]. For a pathogen like smallpox that has been eradicated in the 70s, one can assume that such memory B cells have not been boosted by their cognate antigen since the time of vaccination. It is also very unlikely that anti-vaccinia memory B cells could be boosted by cross-reacting orthopoxviruses since we have never observed any positive reaction against vaccinia virus with memory B cells of young, non-vaccinated people (Huetz et al., in preparation).

What can we learn from these long-lived memory B cells as far as their generation and behavior in front of a boost or a persisting antigen? V_H_ genes in long-lived anti-vaccinia memory B cells carried on average 10–20 mutations, implying that this may reflect the formation of the memory pool during the vaccination protocol (Huetz et al., in preparation). In a recent report on memory B cells from individuals who survived the 1918 influenza pandemic, and taking into account that these memory B cells were most probably boosted by the 1930 cross-reacting influenza infection, their V_H_ genes displayed up to 32 mutations [28]. In the case of a persistent infection such as HIV, up to 60 mutations per V_H_ gene could be observed in HIV-specific memory B cells [29]. It seems therefore that, as in mice, humans generate their memory pool at the beginning of the response with a frequency of mutations in the lower range as compared to GC centroblasts. Upon a boost, which can happen some years after, memory B cells display a higher frequency of mutations implying either that ongoing GCs have continuously fed hypermutated B cells into the memory pool and/or that IgM (and possibly some IgG) memory B cells have gone back to GCs to undergo a new round of SHM [30].

The fact that these memory B cells can remain in the spleen throughout life ready to react to their cognate antigen remains a puzzling biological phenomenon. It has been proposed, according to their in vitro properties, that memory B cells could proliferate in vivo and give rise to plasmocytes under bystander stimulations generated by unrelated immune responses [31]. It is difficult to imagine that such long-lasting memory could be maintained for a lifetime by constant stimulation, considering the number of cell divisions this would imply. Conversely, one could imagine for these memory B cells a quiescent state in a privileged niche in which they would be protected from the surrounding milieu. This status could then be reminiscent of what has been proposed for naive T cells in which an inaccessible compact chromatin prevents any cytokine stimulation unless the antigen receptor is specifically stimulated [32].

## Human IgM B cells with a mutated Ig receptor: marginal zone B cells, IgM memory or B1 cells?

The human and mouse B cell compartment are not easily comparable, with species-specific differences in terms of surface markers (e.g., CD38, CD27) and proportion of effector subsets. Among both splenic and blood B cells in humans, around 70 % of cells are naive CD27^−^ B cells and 30 % are CD27^+^ B cells which are composed equally of switched B cells (15 %) and IgM^+^IgD^+^ B cells (15 %), with IgM-only B cells representing a minor population (1 %) [33, 34].

The marginal zone (MZ) B cell population could be a paradigmatic example of such divergences. In mice, it is widely accepted that MZ B cells are a separate lineage, along which immature B cells engage following a complex array of signals: among those are signals triggered by the Notch2 pathway, tonic signaling from the BCR, signals mediated by survival factors and through distinct NF-κB activators (e.g., the MALT1-Bcl10-CARD11 complex) [35]. Mouse MZ B cells bear an unmutated BCR, reside in the splenic marginal zone and do not recirculate, and respond rapidly to blood borne T-independent antigens, giving rise to IgM and IgG3 antibody-secreting cells. In humans, IgM^+^IgD^+^CD27^+^ B cells present a similar surface phenotype (IgM^high^IgD^low^CD21^high^CD23^−^CD1c^high^) and reside in the marginal zone of the spleen, but they carry a mutated Ig receptor and are recirculating [36].

The presence of somatic mutations in the Ig genes of this subset has nevertheless fostered an alternate interpretation on the origin of IgM^+^IgD^+^CD27^+^ B cells, which would accordingly be the result of a T-dependent response and represent IgM memory B cells that exited the germinal center reaction prior to isotype switch. We have reviewed recently the developmental and functional characteristics of human marginal zone B cells that led us to propose that they may constitute a separate B cell diversification pathway [34], and, on the other side, a thorough critical evaluation of these arguments has been developed by Tangye and colleagues [37].

### Molecular footprints of a germinal center experience in the IgM^+^IgD^+^CD27^+^ B cell subset

Recently, a paper from the group of R. Küppers has brought solid arguments for the existence of a germinal center-derived IgM B cell population in human adult blood [38], which, together with the parallel description by our group of long-lasting antigen-specific IgM B cells in the mouse, strongly shifted the balance toward the IgM memory proposition. In this paper, a thorough analysis of clonal relationships through CDR3-specific amplification identified around one tenth of randomly selected V-D-J junctions from switched B cells as being present within the IgM^+^IgD^+^CD27^+^ compartment, based on a sample size that represent 1–2 % of total blood. The second argument was the presence of mutations in the *BCL6* gene in IgM^+^IgD^+^CD27^+^ cells, a gene activated during the germinal center reaction and preventing the premature detection of AID-induced DNA lesions through transcriptional repression of DNA damage response genes [39]. However, whatever the site where diversification of the IgM^+^IgD^+^CD27^+^ subset may take place, it is not unreasonable to assume that a similar BCL6-mediated control might be required outside the strict germinal center response to allow for mutation accumulation and to prevent DNA damage-induced apoptosis.

While these data obviously establish a direct filiation between (at least some) IgM^+^IgD^+^CD27^+^ and switched B cells in adult blood, should we conclude that the existence of an IgM memory compartment accounts for all the properties of the IgM^+^IgD^+^CD27^+^ subset, both in spleen and blood, in adults and in infants? We would like to briefly summarize some of the recent data, most of which concern developmental/differentiation aspects, which clearly document a distinct behavior of the IgM^+^IgD^+^CD27^+^ subset compared to switched memory B cells.

### Distinct repertoire diversification of the various B cell subsets during B cell ontogeny in infants

**Fig. 3:**
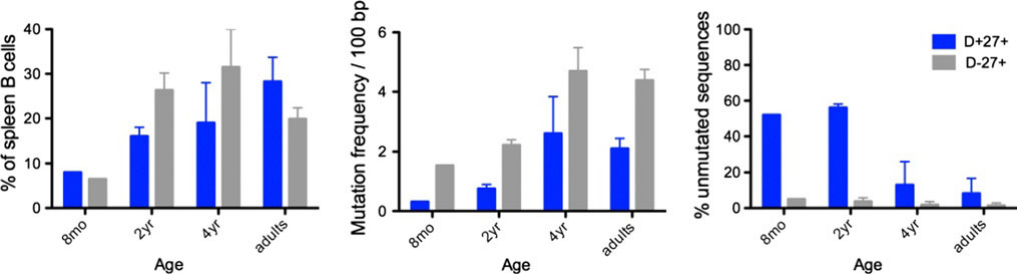
Ontogeny of spleen CD27^+^ subsets and Ig gene mutations. Spleen samples from one infant at 8 months, three children around 2 years of age and two around 4 years, together with three adults, were analyzed for the proportion of IgM^+^IgD^+^CD27^+^ (D^+^27^+^) and switched (D^−^27^+^) B cell subsets among total splenic B cells, the Ig mutation frequency (estimated on a 341 bp fragment from the rearranged J_H_4–J_H_5 intron), and the proportion of unmutated sequences among them ([40] and Descatoire et al., in preparation)

**Fig. 4:**
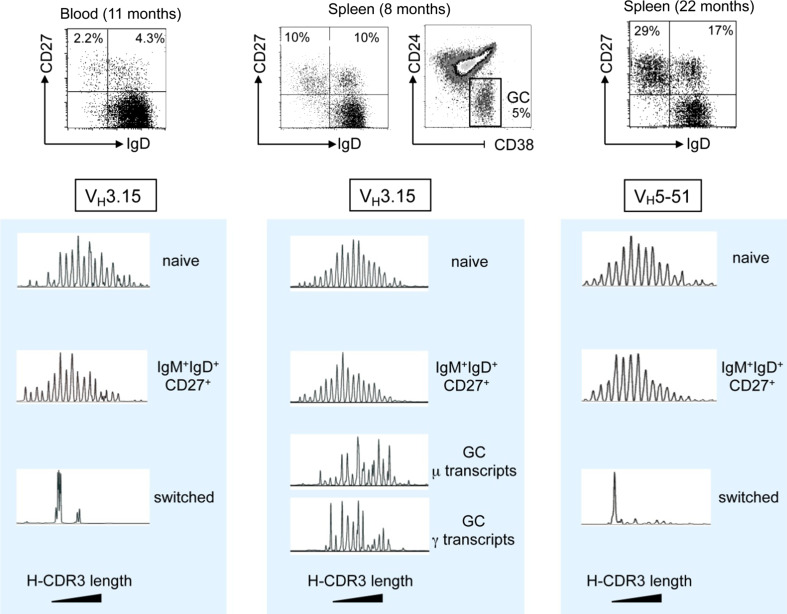
Heavy-chain CDR3 spectratyping of blood and splenic subsets from young children. Blood and spleen samples at the indicated ages were sorted into naive (IgD^+^CD27^−^), IgM^+^IgD^+^CD27^+^, and switched (IgD^−^CD27^+^) subsets, as well as into germinal center (GC) B cells (CD24^−^CD38^+^) for the 8-months spleen sample. Spectratyping for the V3-15 or the V5-51 genes was performed using μ or γ primers, according to the subset analyzed. Increasing CDR3 sizes are represented from left to right. A marked reduced diversity in CDR3 sizes is observed for switched cells and is already apparent at the IgM^+^ germinal center stage, which was confirmed by sequencing for some CDR3 peaks [40]. In spite of a large size distribution similar to naive cells, IgM^+^IgD^+^CD27^+^ cells always display a 1–2 amino acids shift in average CDR3 sizes, a phenotype also described for mouse marginal zone B cells [60]

IgM^+^IgD^+^CD27^+^ and switched B cells develop in parallel in infants (Fig. 3 and [34]). T-dependent germinal center responses appear soon after birth, while immune responses to T-independent antigens remain defective during the first years of life. Up to 3 years of age, germinal center B cells can represent up to 20 % of the total splenic B cell population, a value indicative of the strong immune activation induced by childhood vaccinations [34]. We took advantage of this functional dissociation to analyze the complexity of the repertoire of various B cell subsets from blood or spleen of young children. Using CDR3 spectratyping and sequencing of specific V_H_ rearrangements with defined CDR3 sizes, we were able to show that the repertoire of IgM^+^IgD^+^CD27^+^ B cells was very large, like the one of naive B cells, and displayed no clonal amplification, hallmarks of antigen-induced activation and selection, whether mutated or unmutated sequences were analyzed (see below) (Fig. 4) [40]. By contrast, the repertoire of switched B cells, either in blood or in spleen, appears much more restricted. The presence of abundant germinal centers in these spleen samples allowed the specific analysis of GC B cells, both at the switched and at the early IgM expressing stages (Fig. 4). Most strikingly, repertoire restriction and clonal amplification were already manifest at the IgM^+^ germinal center stage, making unlikely a direct filiation between a population displaying antigen-mediated selection and a population that lacks such imprint [40].

A marked difference was also observed in the ontogeny of mutations between splenic IgM^+^IgD^+^CD27^+^ and switched B cells (Fig. 3) [40]. While most of Ig sequences from switched B cells harbored mutations, about half of the IgM^+^IgD^+^CD27^+^ V_H_ sequences remain unmutated during the first 2 years of life, revealing a much slower accumulation of mutations during early development, in spite of the early acquisition of a CD27 marker.

### Distinct ontogeny of IgM^+^IgD^+^CD27^+^ B cells during recovery from B cell depleting therapies

B cell depleting therapies mediated by anti-CD20 antibodies (Rituximab) are now used to treat a number of autoimmune diseases. Such treatments allowed the follow-up of B cell recovery in “milder” conditions as compared to the treatment of lymphomas that include a general aplasia induced by the drastic concurrent chemotherapy. Such analyses have delineated new intermediates in the B cell maturation pathway (e.g., a “T3” transitional stage) [41]. They have also revealed a slow recovery of the CD27^+^ B cell fraction in the blood, persisting several years after treatment [42, 43]. Interestingly, this delayed reconstitution appeared more pronounced for the IgM^+^IgD^+^CD27^+^ subset, and affected as well the Ig mutation frequency that failed to reach the level of healthy adults over the 6-year period of the study [43, 44]. It remains to uncover what are the factors affecting the B cell recovery process: the presence of a mature adult microenvironment, a lower impact of antigenic challenges mediated by vaccinations, a long-lasting disturbance of immune interactions generated by previous treatments, like corticoids, or even a protracted impact of the anti-CD20 depleting agent. It nevertheless establishes that, like during normal ontogeny, the acquisition of somatic mutations in the IgM^+^IgD^+^CD27^+^ subset is substantially dissociated from those accumulating in switched memory B cells.

### A role for Toll-like receptors in the ontogeny and homeostasis of IgM^+^IgD^+^CD27^+^ B cells

The marginal zone lineage branches at the transitional/immature stage of B cell development, whereas IgM-memory B cells are formed during activation of naive B cells. Previous works from the groups of G. Kelsoe and T. Imanishi-Kari have shown that inflammation and/or TLR signals can drive a T-independent proliferation and accumulation of somatic mutations in immature B cells, which were shown to express a basal level of AID [45, 46]. A similar observation was made recently by R. Carsetti and colleagues for human B cells: activation of immature B cells from cord blood through TLR9 signaling was shown to induce plasmacytic differentiation and isotype switch, as well as proliferation of a subset of these cells accompanied by the acquisition of marginal zone markers (CD27^+^, CD24^+^, CD38^−^) and a low level of somatic mutation [47, 48]. Like in the mouse model, these authors proposed that signals triggered by external bacterial antigens may drive an innate-like response of Ig secretion and early Ig gene diversification after birth, creating a first line of defense against invading bacteria. These cells would then throughout life ensure protection against further infections. Whereas the relevance of this process to in vivo B cell differentiation remains to be demonstrated, it highlights anyway a striking T-independent diversification pathway outside classical germinal center activation.

We have taken a different approach to study the role of TLR signals in the human B cell lineage, through the analysis, in collaboration with the group of J.-L. Casanova, of patients deficient for essential mediators of the TLR signaling pathway: MyD88, IRAK4 (which both affects signaling through all TLRs except TLR3), and Unc93B (which only affects the proper endosomal localization of nucleic acid sensors, TLR3, 7, 8 and 9) [49–51]. A marked reduction of IgM^+^IgD^+^CD27^+^ B cells in presence of a normal switched subset was observed in the case of IRAK4- and MyD88-deficient children and young adults while no specific alteration was observed in Unc93B-deficient patients, with normal Ig mutation frequencies in all cases (Weller et al., submitted). The role of TLR9 thus appears facultative, pointing to the role of other TLRs, like possibly the B cell-specific TLR10, in this deficit. The fact that this deficit was not compensated as patients aged, suggest a role of TLRs in the specific homeostatic maintenance of IgM^+^IgD^+^CD27^+^ B cells. Interestingly, it also indicates that, in spite of the recent implication of MyD88 in a TACI-mediated isotype switch process, MyD88- or IRAK4-deficiency does not impact the peripheral pool of IgG^+^ and IgA^+^ memory B cells in vivo [52].

### Distinct repertoire features revealed by a deep-sequencing analysis of the different B cell subsets from adult blood

A high throughput analysis of V_H_ sequences from blood samples of three adult donors was reported recently [53]. No clonal relationships were found among 1700 sequences from IgM^+^IgD^+^CD27^+^ B cells and 2,000 from switched IgG^+^ and IgA^+^ B cells. Even though the sample size analyzed was very large, this sequencing obviously lacked the sensitivity of the approach developed by R. Küppers and colleagues. Nevertheless, clonal relationships within each subset were recurrently found, with larger and more frequent clonal amplifications among switched B cells. The most striking difference observed concerned the V_H_ repertoire, with a distinct V_H_ usage affecting most importantly the V_H_1 and V_H_3 families: a twofold higher ratio for the V_H_3 and a 20-fold lower ratio for V_H_1 for IgM^+^IgD^+^CD27^+^ B cells compared to switched cells. Such a marked V_H_ bias, together with other minor differences, indicates a minima that different selective forces are shaping the repertoire of these two subsets.

### A human B1 cell compartment?

A newcomer among human B cell subsets is the B1 cell compartment, a long-searched equivalent of the mouse B1 cell lineage present in the spleen and the peritoneal cavity and which is responsible for most of the spontaneous IgM production in this species. This subset, identified through the CD43 marker, and being IgD^+^CD27^+^, displayed B1 cell characteristics, i.e., spontaneous BCR tonic signaling, a low level of spontaneous IgM plasmocyte production, and a capacity to drive allogenic T cell proliferation [54]. Curiously enough, this subset was shown to comprise around 40 % of CD27^+^ B cells in adults, while declining in the elderly, a proportion quite similar to the one of IgM^+^IgD^+^CD27^+^ cells. These figures raised the question as to whether B1 cells and IgM^+^IgD^+^CD27^+^ B cells were simply superimposable, which would have united the human equivalent of mouse circulating B1 and splenic marginal zone B cells in the IgM^+^IgD^+^CD27^+^ compartment as a unique subset with innate-like properties [55, 56]. However, serious concerns have been formulated recently about the effective size of this human B1 population, and, consequently about its intrinsic properties [57, 58].

In conclusion, marginal zone, IgM memory, or B1 B cells? Major differences between classical memory and IgM^+^IgD^+^CD27^+^ B cells remain, concerning mainly their ontogeny and the signals mediating their selection/survival. Later on in adult life, the identity and possible heterogeneity of the IgM^+^IgD^+^CD27^+^ subset, be it in blood or in spleen, obviously appears as an unsettled issue.
